# An mHealth Text Messaging Program Providing Symptom Detection Training and Psychoeducation to Improve Hypoglycemia Self-Management: Intervention Development Study

**DOI:** 10.2196/50374

**Published:** 2023-10-03

**Authors:** Yu Kuei Lin, James E Aikens, Nicole de Zoysa, Diana Hall, Martha Funnell, Robin Nwankwo, Kate Kloss, Melissa J DeJonckheere, Rodica Pop-Busui, Gretchen A Piatt, Stephanie A Amiel, John D Piette

**Affiliations:** 1 Department of Internal Medicine University of Michigan Ann Arbor, MI United States; 2 Department of Family Medicine University of Michigan Ann Arbor, MI United States; 3 Department of Diabetes King’s College Hospital NHS Foundation Trust London United Kingdom; 4 Department of Learning Health Sciences University of Michigan Ann Arbor, MI United States; 5 Department of Diabetes King's College London London United Kingdom; 6 Healthcare System Center for Clinical Management Research VA Ann Arbor Ann Arbor, MI United States; 7 Department of Health Behavior and Health Education University of Michigan Ann Arbor, MI United States

**Keywords:** behavioral intervention, CGM, continuous glucose monitor, design, develop, development, diabetes, diabetic, glucose, hypoglycemia, hypoglycemic, messaging, mHealth, mobile health, self-management, SMS text message, text message, type 1 diabetes, type 1, user-centered

## Abstract

**Background:**

Hypoglycemia remains a challenge for roughly 25% of people with type 1 diabetes (T1D) despite using advanced technologies such as continuous glucose monitors (CGMs) or automated insulin delivery systems. Factors impacting hypoglycemia self-management behaviors (including reduced ability to detect hypoglycemia symptoms and unhelpful hypoglycemia beliefs) can lead to hypoglycemia development in people with T1D who use advanced diabetes technology.

**Objective:**

This study aims to develop a scalable, personalized mobile health (mHealth) behavioral intervention program to improve hypoglycemia self-management and ultimately reduce hypoglycemia in people with T1D who use advanced diabetes technology.

**Methods:**

We (a multidisciplinary team, including clinical and health psychologists, diabetes care and education specialists, endocrinologists, mHealth interventionists and computer engineers, qualitative researchers, and patient partners) jointly developed an mHealth text messaging hypoglycemia behavioral intervention program based on user-centered design principles. The following five iterative steps were taken: (1) conceptualization of hypoglycemia self-management processes and relevant interventions; (2) identification of text message themes and message content development; (3) message revision; (4) patient partner assessments for message readability, language acceptability, and trustworthiness; and (5) message finalization and integration with a CGM data–connected mHealth SMS text message delivery platform. An mHealth web-based SMS text message delivery platform that communicates with a CGM glucose information-sharing platform was also developed.

**Results:**

The mHealth SMS text messaging hypoglycemia behavioral intervention program HypoPals, directed by patients’ own CGM data, delivers personalized intervention messages to (1) improve hypoglycemia symptom detection and (2) elicit self-reflection, provide fact-based education, and suggest practical health behaviors to address unhelpful hypoglycemia beliefs and promote hypoglycemia self-management. The program is designed to message patients up to 4 times per day over a 10-week period.

**Conclusions:**

A rigorous conceptual framework, a multidisciplinary team (including patient partners), and behavior change techniques were incorporated to create a scalable, personalized mHealth SMS text messaging behavioral intervention. This program was systematically developed to improve hypoglycemia self-management in advanced diabetes technology users with T1D. A clinical trial is needed to evaluate the program’s efficacy for future clinical implementation.

## Introduction

Hypoglycemia episodes, especially those with blood glucose <54 mg/dL, can cause acute problems including brain dysfunction [1,2] and cardiac arrhythmias [3]. The condition is also associated with longer-term complications such as memory and psychomotor function decline [4,5], adverse cardiovascular events [6,7], and increased mortality [8,9]. People with type 1 diabetes (T1D) are particularly susceptible to hypoglycemia due to the use of exogenous insulin and the loss of hypoglycemia-induced glucagon secretion [10]. Antecedent hypoglycemic episodes with blood glucose <54 mg/dL also exacerbate hypoglycemia risks by reducing patients’ ability to detect hypoglycemia symptoms (also known as impaired awareness of hypoglycemia [11]) and compromising autonomic counterregulatory responses [12]. The worldwide prevalence of T1D has doubled in the past 2 decades [13] and is expected to reach 15 million people in 2040 [14]. Scalable interventions to minimize hypoglycemia are thus critically needed.

Several technologies developed over the past decade have led to major breakthroughs in diabetes management, including in reducing hypoglycemia [15]. In particular, continuous glucose monitors (CGMs) provide users with real-time glucose information for timely hypoglycemia recognition and self-management [16]. Automated insulin delivery (AID) systems use algorithms together with real-time CGM glucose information to automatically change or suspend basal insulin doses on insulin infusion pumps [17]. CGMs and AID systems have been shown to reduce hypoglycemia in clinical trials [18-21] and observational studies [22-24]. These advanced diabetes technologies are now the standard of care for people with T1D [25], and the adoption rate of CGMs has increased from 6% in 2011 to 38% in 2018 [26] and 60% in 2022, according to reports of large-scale observational studies [27]. Since AID systems became commercially available in the United States in 2016, the rate has remained as low as 7%, based on 2022 national registry data [28].

Even with these innovations, data from clinical trials [29-31] and from institutional [32,33] and national patient registries [23,34] indicate that about a quarter of people with T1D who use advanced diabetes technologies continue to spend ≥1% of time with glucose <54 mg/dL (ie, above the recommended target [35]) or develop severe hypoglycemia (ie, episodes requiring assistance from other people for management [36]). Research using survey [31,32,37,38] and qualitative [39,40] methods has indicated that hypoglycemia symptom detection remains integral to hypoglycemia self-management in people with T1D who use advanced diabetes technology. Furthermore, unhelpful hypoglycemia beliefs may delay or prevent hypoglycemia self-management [33,39,40]. Programs that improve hypoglycemia symptom detection and address unhelpful beliefs have been found to reduce this state in cohorts including advanced diabetes technology users [41,42]. A scalable, effective behavioral intervention targeting people with T1D who use advanced diabetes technology is urgently needed to optimize the use of such technologies while minimizing hypoglycemia in this population [43].

Mobile health (mHealth) uses mobile communication (eg, apps and SMS text messages [44]) to deliver scalable interventions to patients in their everyday living environment to improve clinical outcomes [45-47]; mHealth has demonstrated effectiveness in delivering psychological interventions [48,49] and enhancing chronic disease self-management [45,50] as measured by self-care, perceived self-efficacy, and glucose control in people living with diabetes [51-58]. More than 5 billion people worldwide use mobile technologies. Therefore, mHealth could be a scalable, accessible alternative to in-person clinical visits for certain interventions [59]. All major CGM brands currently have smartphone apps enabling patients to access their personal glucose data and share that information with clinicians and other parties as indicated. A recent survey revealed that 98% of T1D advanced diabetes technology users actively use smartphones, with 80% of this population being open to receiving mHealth interventions (including 98% who would be willing to share CGM data) for better glucose management [60]; mHealth could thus be a vehicle for hypoglycemia prevention in people with T1D, including those who use advanced diabetes technologies.

This formative research was intended to develop a scalable, personalized mHealth behavioral intervention program, HypoPals, focusing on reducing hypoglycemic episodes, particularly those with blood glucose <54 mg/dL, among people with T1D who use CGMs.

## Methods

### Overview

**Figure 1:**
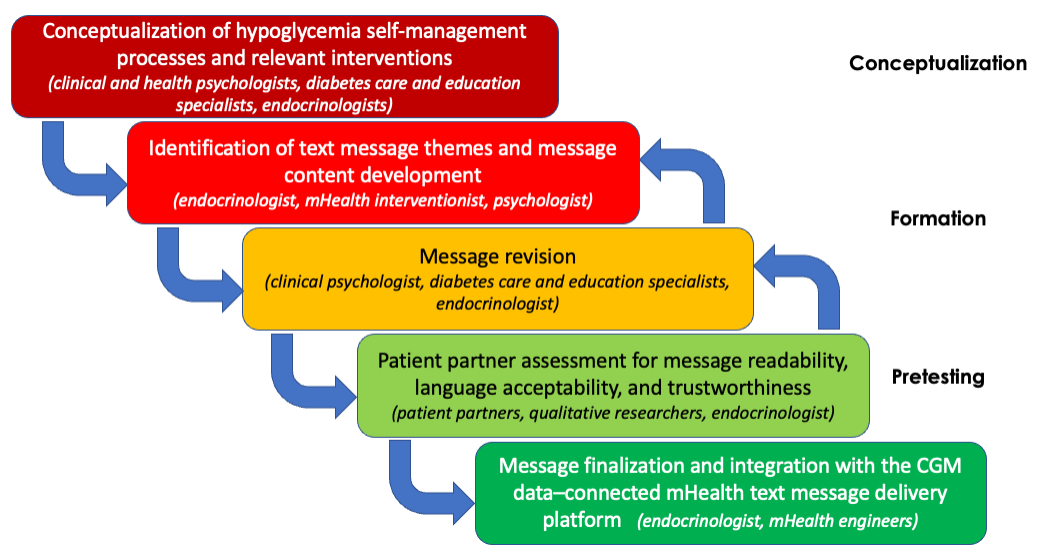
Steps in HypoPals intervention message development. CGM: continuous glucose monitor; mHealth: mobile health.

A multidisciplinary team (including clinical and health psychologists, diabetes care and education specialists, endocrinologists, mHealth interventionists, computer engineers, qualitative researchers, and patient partners) convened to develop the HypoPals mHealth SMS text messaging behavioral intervention program. User-centered design principles [61] were adopted and operationalized into iterative steps for program creation: (1) conceptualizing hypoglycemia self-management processes and relevant interventions; (2) identifying text message themes and drafting content for individual messages; (3) revising messages; (4) evaluating message readability, language acceptability, and trustworthiness with patient partners; and (5) finalizing messages for integration with an mHealth SMS text message delivery platform connected to users’ CGM glucose data. Figure 1 depicts these steps and their associated contributors. The Twilio platform [62] was used to build the mHealth web-based SMS text message delivery platform that communicated with the CGM glucose information-sharing platform through an application programming interface.

### Step 1: Conceptualization of Hypoglycemia Self-Management Processes and Relevant Interventions

**Figure 2:**
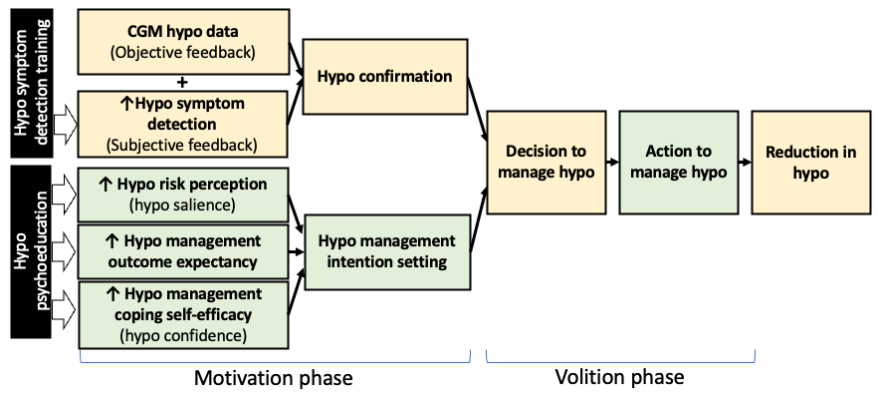
Conceptual framework of hypoglycemia self-management in people with type 1 diabetes who use advanced diabetes technologies and relevant interventions. This conceptual framework integrates constructs adapted from the biopsychobehavioral model of severe hypoglycemia (yellow boxes) and the health action process approach model (green boxes). Interventions for improving hypoglycemia self-management and reducing hypoglycemia are presented in black boxes. CGM: continuous glucose monitor; hypo: hypoglycemia.

We previously conducted survey [32,37] and qualitative [39,40] studies that recruited participants ranging from those who spent a minimal amount of time with hypoglycemia to those who frequently developed severe hypoglycemic episodes; we identified that hypoglycemia symptom detection supports patients’ confirmation of CGM hypoglycemia information and facilitates self-management. Unhelpful hypoglycemia beliefs that can contribute to poor self-management include the following: minimal hypoglycemia risk perceptions; high perceptions of hyperglycemia and social risks from hypoglycemia self-management (which can lead to low hypoglycemia self-management outcome expectancies); and low hypoglycemia management coping efficacy [33,39,40]. Importantly, previous research suggested that impaired hypoglycemia awareness and having unhelpful hypoglycemia beliefs do not necessarily interact, and thus they may independently contribute to hypoglycemia development [33]. Based on this, constructs from the biopsychobehavioral model of severe hypoglycemia [63] and the health action process approach model [64] were adapted to reflect the theoretical construct that hypoglycemia symptom detection and hypoglycemia beliefs play independent key roles in hypoglycemia self-management in CGM users with T1D (Figure 2). Hypoglycemia symptom detection training could improve symptom detection and hypoglycemia confirmation [41], while hypoglycemia psychoeducation could address unhelpful beliefs and promote hypoglycemia management intention setting [42]. Both interventions are expected to lead to decisions and actions that promote hypoglycemia self-management and therefore reduce hypoglycemia.

### Step 2: Identification of Text Message Themes and Message Content Development

To develop hypoglycemia symptom detection training messages, concepts from blood glucose awareness training (eg, careful self-assessment of hypoglycemia symptoms improves hypoglycemia symptom detection) [65] were reviewed and adapted to develop text message themes. To compose messages for addressing unhelpful hypoglycemia beliefs, concepts and materials from the Hypoglycemia Awareness Restoration Program for People With Problematic Hypoglycemia Despite Optimised Care (HARPdoc) [66], a psychoeducation program targeting unhelpful hypoglycemia beliefs for reducing hypoglycemia, were also integrated to develop a list of psychoeducation message themes.

During the message drafting, message series focusing on separate themes were jointly compiled by an endocrinologist (YKL) and an mHealth interventionist and health psychologist (JEA). Rigorous discussions were held regarding concepts, writing styles, behavior change strategies, and message length and reading level when creating each message series. Experiences with hypoglycemia and hypoglycemia self-management from people living with T1D (including practical behavioral solutions) were incorporated into the messages. Two-way interactive messaging techniques were incorporated for increasing the engagement with and the efficacy of intervention messages [67]. Weekly messages that summarize week-based hypoglycemia outcomes were also developed for patients to self-review behaviors that are perceived as helpful or unhelpful for their hypoglycemia self-management. In line with the concept of HARPdoc, the psychoeducation messages integrated behavioral change techniques, including motivational interviewing (eg, decision matrix) and cognitive behavioral therapy techniques to challenge beliefs using Socratic-style questions, problem-solving, and exposure [66]. All text messages were written in a friendly, autonomy-respecting, and nonjudgmental tone [68].

### Step 3: Message Revisions

An endocrinologist (SAA) and a diabetes-specialized clinical psychologist (NDZ), 2 HARPdoc developers, individually reviewed the messages and suggested revisions. Messages were then edited by the message developers (JEA and YKL) and rereviewed by SAA and NDZ for additional input and modification as needed. A diabetes care and education specialist team (MF, RN, and KK) also met with the message developers to discuss and iteratively revise each message. Conversations focused on the message content, consistency with current diabetes education materials, readability, language acceptability, and use of behavior change techniques. The revision team offered more feedback during the revisions with patient partners (described below) until no further concerns or suggestions were raised.

### Step 4: Patient Partner Assessments for Message Readability, Language Acceptability, and Trustworthiness

To ensure user-level message readability, language acceptability, and trustworthiness [69], 14 focus group interviews were held with 31 patient partners (ie, people living with T1D and using CGMs; DH was the moderator and YKL was the notetaker). An endocrinologist (YKL) and qualitative researcher (MJD) codeveloped the interview guide (Textbox 1). Focus group interviews were held through Zoom due to the COVID-19 pandemic. The small number of respondents (2-4) enabled patient partners to review the message content carefully and provide prompt feedback without impeding the patient partners’ conversations. Participants received draft message series through cell phone to approximate real-life experiences; participants were then asked to read the messages and provide feedback regarding readability, language acceptability, and trustworthiness. The focus group interview guide evaluated participants’ understanding of each message, general impressions, potential bothersome language or content, and messages’ trustworthiness [68]. Sampling and participant characteristics are reported in Multimedia Appendix 1.

**Focus group interview guide.**

**Interview guide**

- To you, what were the messages trying to say? (to determine if messages were perceived as intended)
- How do you think of the messages in general?
- What are the things you don’t like as much about how the messages were written?
- Do you trust what the messages were telling you?
- What else came to your mind when reading these messages?

Each focus group convened for up to 2 hours to ensure participants remained engaged in the web-based interview. Each patient partner could only attend up to 2 focus group interviews to prevent feedback from a small portion of respondents skewing the evaluation and revision processes, which would limit the messages’ generalizability. All draft messages were reviewed, and about 70% were edited for phrasing or content based on patient partners’ suggestions. All revised messages were then rereviewed with other patient partners to enhance generalizability.

### Step 5: Message Finalization and Integration With a CGM Data–Connected mHealth SMS Text Message Delivery Platform

An mHealth web-based SMS text messaging platform that communicates with the Dexcom CGM glucose information-sharing platform was developed using Twilio [62]. Databases (eg, HypoPals program users’ telephone numbers and emails; delivered interventional text message content; and delivery timestamps) were maintained on secure servers at the University of Michigan Intelligence Transportation System. Patient CGM data transfers and data use authentication were completed through Dexcom’s CGM glucose information-sharing platform, Dexcom Developer [70]. Transfers of actual CGM data occurred through the same platform. The developed mHealth application programming interface received all CGM data and used that data to tailor text messages. The data were then applied to calculate users’ time in hypoglycemia for either directing weekly CGM summary messages or triggering the delivery of hypoglycemia symptom detection messages, which are to be sent after CGM-detected hypoglycemic episodes (ie, CGM glucose <70 mg/dL lasting ≥15 minutes [71]). During the finalization stage, the mHealth technology team reviewed the intervention messages before they were incorporated into the mHealth SMS text message delivery platform. The messages were then reviewed again to ensure proper content and order of delivery.

Dexcom CGMs were used in this phase of HypoPals development because Dexcom Developer is a Food and Drug Administration–approved platform for securely sharing patients’ CGM data with third-party app developers [70]. The high use rate of Dexcom CGMs in the United States [26,72] at the time when the study was planned could support future efficacy studies as well as future HypoPals implementation. HypoPals development aimed to maximize the generalizability of message content (ie, by creating content suitable for general advanced diabetes technology users rather than solely Dexcom users).

### Ethics Approval

The University of Michigan Institutional Review Board approved this study (HUM00184315, HUM00221736).

## Results

### Overview

A CGM data–directed mHealth SMS text messaging program, HypoPals, was created to enhance hypoglycemia self-management and reduce hypoglycemia in people with T1D who continue to develop severe hypoglycemia or spend time with glucose <54 mg/dL above the recommended target [35] despite using advanced diabetes technologies. HypoPals delivers intervention messages to promote hypoglycemia symptom knowledge and offers opportunities to practice and potentially improve hypoglycemia symptom detection. The psychoeducation messages were designed to elicit self-reflection, provide fact-based education, and suggest practical health behaviors to address unhelpful hypoglycemia beliefs and promote hypoglycemia self-management. Message series for hypoglycemia symptom training were delivered within 3-15 hours following a hypoglycemic episode (up to 3 series per day). Hypoglycemia psychoeducation messages were delivered once daily; specifically, between 1 and 4 message series were delivered each day throughout the 10-week intervention.

### Hypoglycemia Symptom Detection Training

**Figure 3:**
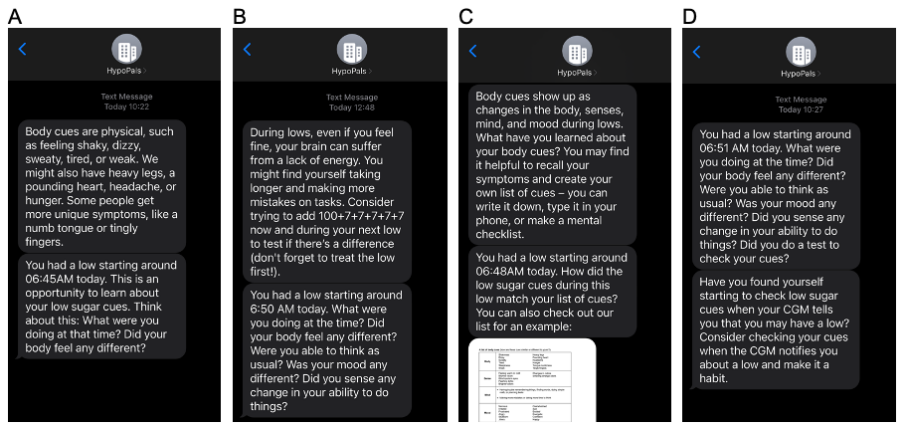
Examples of HypoPals hypoglycemia symptom detection training messages (A) describing hypoglycemia symptoms and self-checks; (B) describing a self-test to detect mental changes during hypoglycemia; (C) encouraging patients to develop an understanding of their own hypoglycemia symptoms; and (D) encouraging patients to develop a habit of self-checking hypoglycemia symptoms upon receiving continuous glucose monitor hypoglycemia data.

Hypoglycemia symptom detection training includes 27 message series. Each series typically contains a pair of messages. The first message in each series normally presents information about hypoglycemia symptoms and outlines symptom detection behavior in a stepwise fashion by covering (1) the importance and use of hypoglycemia symptoms as biological feedback for decision-making; (2) types of hypoglycemia symptoms, such as bodily sensations or sensory, mental, and mood changes, along with quick self-tests that patients can perform to detect psychological changes during hypoglycemia; (3) creation of a list of personal hypoglycemia symptoms; and (4) the need to routinely self-check hypoglycemia symptoms upon receiving CGM hypoglycemia data. The second message in the series generally indicates the time of a recent CGM-detected hypoglycemic episode and encourages users to recall their hypoglycemia symptoms at that time. All messages are meant to encourage habitual self-checking of hypoglycemia symptoms when patients receive CGM information about ongoing hypoglycemia. Sample messages are displayed in Figure 3. The symptom self-checking messages continue until week 10 (when the program ends) or until the patient requests that the program stop sending messages.

The mHealth SMS text messaging technology scans for CGM hypoglycemic episodes (ie, glucose level <70 mg/dL consecutively for ≥15 minutes [71]) 3 times a day (10 AM, 3 PM, and 10 PM; time-zone sensitive). The system sends messages when at least one hypoglycemic episode is detected between each scan.

### Hypoglycemia Psychoeducation

**Figure 4:**
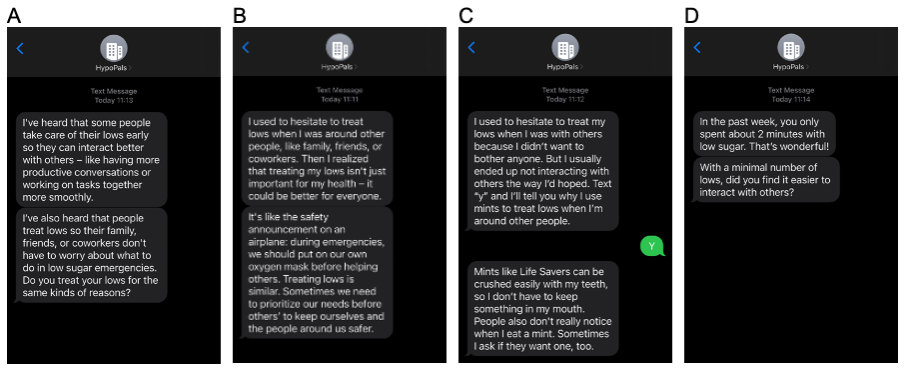
Examples of HypoPals hypoglycemia psychoeducation messages. (A) A message eliciting social benefit–related internal motivation for hypoglycemia self-management; (B) a message illustrating overall benefits of hypoglycemia self-management, including in social situations; (C) 2-way SMS text messages for practical health behaviors focused on hypoglycemia self-management; and (D) a weekly feedback message to promote the social benefits of avoiding hypoglycemia.

HypoPals’s hypoglycemia psychoeducation includes 77 message series. Each series contains between 1 and 3 messages, and 14 series contain an additional 2-way interactive message. The messages are intended to (1) calibrate hypoglycemia risk perceptions, (2) improve hypoglycemia self-management outcome expectancies, and (3) increase hypoglycemia self-management coping efficacy. Methods such as eliciting self-reflection, providing fact-based education, and suggesting practical health behaviors are included in the program to address unhelpful hypoglycemia beliefs and promote hypoglycemia self-management. Chronologically, message topics first entail (1) identifying internal motivations to increase hypoglycemia self-management outcome expectancies and (2) eliciting perceived hypoglycemia risks to mitigate associated risk perceptions. The topics then evolve to focus on (3) addressing strong perceptions of hyperglycemia risks and social risks from self-management to further improve hypoglycemia management outcome expectancies and (4) suggesting practical behaviors related to hypoglycemia self-management to reinforce self-management self-efficacy. Every 7 days, based on the time spent in hypoglycemia, the user receives either an encouraging message or a gentle reminder to promote self-monitoring and hypoglycemia self-management. Sample messages are shown in Figure 4. The psychoeducation messages continue until week 10 (when the program ends) or until the patient requests that the program stop sending messages. Although HypoPals includes 77 developed message series, not all will be delivered to every patient; weekly messages are determined based on a user’s CGM-indicated time in hypoglycemia.

During the focus group interviews with patient partners (ie, step 4), participants generally reported to have found the intervention messages trustworthy. At the early phase of this step, comments about the message readability often focused on the message length or the content itself: “I think this is way too long. I don’t know how to fix it but you better shorten it” and “I don’t understand what this means. Tell me what it was trying to say.” Many patient partners actively offered suggestions on how to improve the readability of the messages: “Perhaps you can shorten this by saying...”; “I don’t know anything about psychology but I will better understand it if you say...”; and “Maybe you should break down these messages to make them shorter.” Critical comments on the acceptability were also made early in the revision phase: “I don’t like how this was written; it makes feel that I am not in control. Maybe you can change this to...” and “This kind of feedback makes me uncomfortable. You should try to make it more positive.” No concerns about readability or acceptability emerged during the later phase of this revision step. Instead, comments from patient partners began to focus on the perceived effectiveness while this concept was not specifically assessed: “This is really, really helpful; you guys are doing a great job!”; “This is giving me goosebumps – it is just talking about me!”; “I am the only person I know who has diabetes, and this is helping me know that there are also others around experiencing what I experience”; and “I wish people [had] told me about [this information] years ago. I probably could’ve lived a better life.”

**Figure 5:**
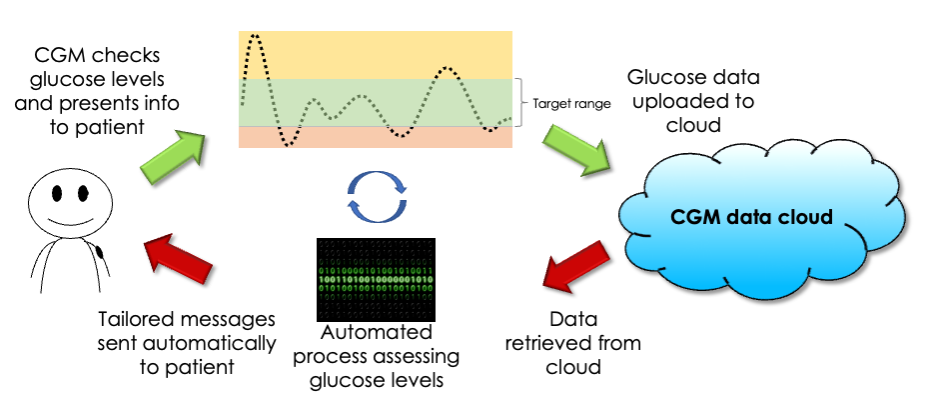
Closed-loop continuous glucose monitor (CGM)–mobile health behavioral intervention SMS text messaging system.

In terms of technological processes, the mHealth platform regularly retrieves users’ CGM glucose data to deliver intervention messages and achieve a closed-loop CGM-mHealth SMS text message intervention cycle (Figure 5) [73].

## Discussion

### Overview

Advanced diabetes technologies have helped reduce hypoglycemia in people living with T1D. However, no widely available interventions exist to further assist patients who continue to develop this dangerous complication. The literature suggests that a lowered ability to detect hypoglycemia symptoms and unhelpful hypoglycemia beliefs hinder hypoglycemia self-management among T1D advanced diabetes technology users. We developed HypoPals, a CGM data–directed hypoglycemia behavioral intervention program, to address these gaps. The program is meant to promote hypoglycemia self-management and ultimately minimize hypoglycemia among people with T1D who use advanced diabetes technologies.

HypoPals has several strengths. First, we developed a detailed conceptual framework grounded in quantitative and qualitative research. The model was adapted from established theories, including a hypoglycemia biopsychobehavioral model [63]. Program intervention strategies were created to target key theory-based facilitators and barriers to hypoglycemia self-management (ie, symptom detection and unhelpful beliefs). Concepts, materials, and evidence-based behavior change strategies were integrated from effective hypoglycemia behavioral interventions [41,66]. mHealth techniques that facilitate effective diabetes self-management, including 2-way interactive messaging and data-based personalized feedback, were also used [57]. Intervention messages reflected patients’ actual experiences with hypoglycemia. Finally, all messages were rigorously evaluated and revised with stakeholders’ input (ie, from clinical and health psychologists, diabetes care and education specialists, and patient partners). The confirmed readability, acceptability, and trustworthiness of messages are expected to enhance the program’s fidelity and effectiveness.

HypoPals is innovative in several ways. To our knowledge, this theory-driven program is the first to adopt mHealth as delivery vehicle for a hypoglycemia behavioral intervention. This technique could promote scalability and consistent delivery (and thus fidelity and reproducibility) in the future [74]. Furthermore, CGM data were integrated to enable automated, timely interventions (eg, hypoglycemia-triggered symptom detection training) and personally relevant feedback (eg, providing the patient’s time in hypoglycemia over the past week to encourage users in either maintaining or adapting their behavior). Such information is intended to increase intervention adherence and impact [57].

Notably, HypoPals was not created to replace conventional diabetes self-management and support programs. People with T1D presumably need comprehensive diabetes self-management training on issues including insulin dosing, meal content, exercise, and fast-acting carbohydrates to adequately process the program’s content. Thus, HypoPals may play an adjunctive role—particularly for patients who continue to experience severe hypoglycemia (eg, frequently have glucose <54 mg/dL) or are at high risk due to impaired hypoglycemia awareness or unhelpful hypoglycemia beliefs despite the current standard of care, including completion of comprehensive diabetes education and using advanced diabetes technologies.

It is important to point out that, while HypoPals was designed to address multiple barriers to hypoglycemia self-management in advanced diabetes technology users, HypoPals alone is unlikely to resolve all hypoglycemia on CGM. For example, social determinants of health, including limited access to food, have been related to hypoglycemia development [75]. While HypoPals may increase the ability to confirm CGM hypoglycemia and the intention to manage hypoglycemia, HypoPals is unlikely to be able to tackle socioeconomic barriers. Similarly, technology-related limitations, including CGM sensor accuracy issues [76] and alarm fatigue, can remain challenging. However, if proven to be effective, HypoPals will likely help patients identify and be more cautious of real hypoglycemic episodes and thus prevent dangerous hypoglycemia. Also, during step 4 message readability revision, education level was not considered during the recruitment of patient experts. The readability of the intervention messages is to be further evaluated with groups with various education levels.

### Future Directions and Vision

HypoPals’s efficacy in reducing hypoglycemia has not been formally tested [77]. The program may affect users differently according to their hypoglycemia-related characteristics, sociodemographics, or social determinants of health. For example, people who cannot always detect hypoglycemia symptoms might especially benefit from symptom detection training messages. Similarly, users with unhelpful hypoglycemia beliefs at baseline may show greater reductions in hypoglycemia after receiving psychoeducation messages. A greater understanding of these potential moderating relationships can reveal ways to further personalize the intervention for each patient.

If this program’s efficacy is confirmed, then further modifications could make HypoPals more scalable. For instance, while the current iteration relies on Dexcom CGM data, the program’s conceptualization and methods could also apply to users of other CGM systems. Moreover, because this intervention was developed with US patient partners, cultural and linguistic adaptations may be needed to deploy it more globally.

### Conclusions

A CGM data–directed mHealth hypoglycemia behavioral intervention program was developed to improve hypoglycemia self-management among people with T1D using advanced diabetes technologies. Efficacy testing is the next step. To date, this theory-based, scalable program has potential for improving hypoglycemia symptom detection and addressing unhelpful hypoglycemia beliefs in order to reduce hypoglycemia. If proven to be effective, HypoPals can lessen hypoglycemia-related complications and burdens among people who continue to struggle with these concerns despite the current standard of care.

## Appendix

Multimedia Appendix 1 Sampling and participant characteristics of the focus group interviews for intervention text message revision.

## Abbreviations

AID: automated insulin delivery system
CGM: continuous glucose monitor
HARPdoc: Hypoglycemia Awareness Restoration Programme for People With Type 1 Diabetes and Problematic Hypoglycemia Persisting Despite Optimised Care
mHealth: mobile health
T1D: type 1 diabetes
