# Supplementary material for: An mHealth Text Messaging Program Providing Symptom Detection Training and Psychoeducation to Improve Hypoglycemia Self-Management: Intervention Development Study
Source: JMIR Form Res. 2023 Oct 3;7:e50374. doi: 10.2196/50374 (PMC10582820; doi:10.2196/50374)
Supplement: Multimedia Appendix 1 [file formative_v7i1e50374_app1.docx]

**Supplement 1 – Sampling and participant characteristics of the focus group interviews for intervention text message revision**

The study was approved by the University of Michigan (U-M) Institute Review Board (HUM#00221736).

Eligibility

Inclusion criteria:

- Diagnosis of type 1 diabetes
- Age ≥18 years old
- Using continuous glucose monitoring system (CGM) for at least 1 year.

Exclusion criteria:

- Uncontrolled psychological conditions or chronic cognitive dysfunction

Sampling strategy

Purposive sampling based on patient age, sex and racioethnic characteristics

Candidate pool and recruitment

The Adult Diabetes Education Program at U-M regularly coordinates with patients, medical device supply companies, insurance companies, and clinicians to initiate and refill advanced diabetes technology supplies for patients at U-M and holds a constantly updated list of patients using these technologies. A list of diabetes technology users at U-M was obtained. After reviewing age, sex and racioethnic information, candidates were selected and recruitment letters/emails were generated and sent. The recruitment letter/email contains a weblink to a Research Electronic Data Capture (REDCap) survey website, which provided information on this focused group interview study and inquired candidates’ interests in participating in the study. Phone calls and voice messages were generated to reach to the candidates who had reported to be interested in the study, and to check eligibility, complete the consent process, and coordinate to schedule focused group interviews.

Supplemental Table 1. Participant characteristics N=31

| **Participant Characteristics** | **Mean±standard deviation (Range) or N (%)** |
| --- | --- |
| Age | 40±16 (21-72) |
| Sex, female | 16 (52%) |
| Race |  |
| Caucasian | 29 (94%) |
| African American | 2 (6%) |
| Asian | 1 (3%) |
| Ethnicity |  |
| Non-Hispanic | 30 (97%) |
| Unknown | 1 (3%) |
